# New era towards advancing in girls’ and women’s health and rights?

**DOI:** 10.1186/s12978-017-0340-3

**Published:** 2017-06-29

**Authors:** José M. Belizán, Suellen Miller

**Affiliations:** 1 Institute for Clinical Effectiveness and Health Policy, Ravignani, 2024 Buenos Aires, Argentina; 2 Safe Motherhood Program, School of Medicine, Department of Obstetrics, Gynecology, and Reproductive Sciences, University of California, San Francisco, USA

Whenever there are leadership changes in the global health sphere, there are always expectations, hopes, and optimism. 2017 ushers in a new era of global leadership at the World Health Organization (WHO) and the UN. All of us involved in making reproductive health more equitable, particularly for the most vulnerable, would like to see profound and rapid changes. During the campaign for the Director General (DG) position at WHO, the candidates responded to a series of questions from the organization, Women Deliver, (http://womendeliver.org/2017/close-personal-three-final-director-general-candidates/).

Dr. Tedros Adhanom Ghebreyesus, the new WHO Director General (DG), made commitments during his campaign, these commitments could result in powerful changes and improved health outcomes. In his talking points candidate Dr. Tedros addressed questions about difficult-to change topics. In relation to gender equality he said: *“I believe that gender equality brings sustainable development – that investments in girls’ and women’s health and rights are investments in a healthy and more prosperous future. Healthy, empowered girls and women have the potential to build stronger communities, economies and nations and ultimately transform entire societies. As Director-General, I will encourage bolder and more sustainable investments and partnerships to advance girls’ and women’s health and rights.”*

On how he would ensure that girls’ and women’s health, rights and wellbeing would be mainstreamed across WHO’s work Tedros stated: “*Women, girls, and young people must be at the center of WHO’s work and mandate – because when they thrive, everybody does. As Director-General, I will re-orient WHO’s approach to focus on women, children and adolescents, particularly those living in humanitarian, fragile and hard-to-reach settings. I will also strengthen WHO’s capacity to monitor results, resources and rights, in line with the goals of the Global Strategy for Women’s, Children’s and Adolescents’ Health* [1]*, and hold governments accountable for their commitments. I will advocate for increased investments, including a Grand Challenges initiative to address key gaps by developing innovations that empower women and girls. And I will continue and intensify WHO’s work with other UN agencies and global stakeholders and initiatives.”*

On the issue about the place that sexual and reproductive health and rights should have on the global health and sustainable development agendas - and in WHO, Tedros stated: *“Family planning is one of the most game-changing interventions for women, families, communities and countries. The increased use of family planning services and birth spacing among women offers increased opportunities to participate in the labor force, which in turn led to economic and political empowerment. We will only be able to realize the ambitious health and development targets set forth by the Sustainable Development Goals if we make sexual and reproductive health and rights a top priority on the global health and sustainable development agendas. WHO must work alongside governments and regional organizations – in close collaboration with civil society, private sector, other UN agencies, donors and other key stakeholders – to drive the global sexual and reproductive health and rights agenda so that women, men and young people can access the full range of sexual and reproductive health services of their choice.”*

Regarding how he would work to ensure that adolescents and young people sexual and reproductive health needs and opportunities are better met, he replied: *“The wellbeing of women, children and adolescents is one of my leadership priorities. Around the world, far too many young people lack access to the sexual and reproductive health services they need to survive, thrive and achieve their full potential. Addressing the unique needs of adolescents will require a multi-sectoral approach. Girls’ education is a strategic solution to many of the challenges that young women face – health, economic, and political. The longer that girls are enrolled in formal education, the more informed and empowered they become in managing their personal health and relationships, including sexual and reproductive health. Providing comprehensive sexual education services in schools and preventing school drop outs particularly due to health issues such as menstrual hygiene should be part of a comprehensive sexual and reproductive health agenda for adolescents. Young girls are becoming brides and mothers too soon, endangering both their health and the fundamental rights. I will champion the implementation and monitoring of the Global Strategy for Women’s, Children’s and Adolescents’ Health* [1]*, as well as the development of stronger and better-resourced national adolescent health and well-being programs, including in schools”.*

Finally, regarding what would his key priorities as Director-General of WHO would be, he mentioned among others: *“During my tenure as Director-General, I will continue to champion gender equality and promise to place the wellbeing of women, children and adolescents at the center of my agenda. Achieving the ambitious targets of the Sustainable Development Goals requires improving the health, dignity and rights of women, children and adolescents. We need to address the lack of access to maternal health, sexual and reproductive health, family planning and adolescent health services. We need innovation, research and data to develop gender-responsive health policies. We also need effective partnerships with national governments, civil society and the private sector to drive progress. Together, we can put health at the center of the gender equality agenda – and gender equality at the center of the health agenda. I will transform WHO into a more effective, transparent and accountable agency that is independent, science- and innovation-based, responsive and harmonized, with a shared vision across all levels. I will advance universal health coverage to ensure all people can access the services they need without risk of impoverishment. This includes leveraging domestic resources for health, strengthening primary health care, and expanding access to sexual and reproductive health services, as well as preventive services, diagnostics and high-quality medicines for communicable and non-communicable diseases. These efforts should identify and scale up best practices as well as tailoring the actions into the needs and context of countries”.*

Taking candidate Tedros’ pre-election commitments into action requires that he immediately implement major changes as Director General. The scientific Reproductive Health (RH) community can be important contributors to achieve many of these commitments. We expect that WHO and UN Agencies will support research initiatives, structures and networking of research institutions in Low and Middle Income Countries. These countries should be the generators of research needs, research focus, global funds’ assignment, and be the implementers of research activities [2]. Making this a strong policy in global RH research would allow for development and testing of interventions that are generated on the local needs and a more realistic feasibility of how to implement projects in local contexts. Ultimately, these actions could lead to a more efficient deployment of research results and better health outcomes.

UN Agencies need to be accountable for meeting these commitments, and should demonstrate that accountability by having external groups evaluate their progress against these commitments, promises, and statements. These evaluations should demonstrate action taken and achievements met [3]. As an example of how this can be done, the Global Strategy for Women’s and Children’s Health 2016–2030 have precise indicators to follow countries’ progress. Likewise, regardless of the fact that UN agencies are composed of national governments, the UN agencies must be independent and have the authority to hold countries accountable for not meeting targets and commitments [4].

We are pleased to hear candidate Tedro’s commitments to Reproductive Health, women’s health, adolescent health, and global equity for vulnerable populations. Now we call on the RH communities to work with Dr. Tedros, WHO, and other agencies to hold them accountable and to turn these verbal commitments into actionable advances in health.
